# Guideline on the Use of Intravenous Ketamine for Procedural Sedation in the Children’s Emergency Department: A Quality Improvement Project

**DOI:** 10.7759/cureus.75085

**Published:** 2024-12-04

**Authors:** Samuel I Udo, Charles Rich, Joshua Lyon

**Affiliations:** 1 Emergency Medicine, Buckinghamshire Healthcare NHS Trust, Buckinghamshire, GBR; 2 Emergency Medicine and Intensive Care Medicine, Buckinghamshire Healthcare NHS Trust, Buckingham, GBR; 3 Emergency Medicine and Pediatric Emergency Medicine, Buckinghamshire Healthcare NHS Trust, Buckingham, GBR

**Keywords:** children's, ed, emergency department, ketamine, procedural sedation challenges, procedure sedation, qip, uk - united kingdom

## Abstract

In pediatric emergency medicine, sedation is crucial for performing some therapeutic procedures in children. Ketamine is still not widely used, despite being the preferred agent due to its effectiveness and safety profile. Implementing a guideline for intravenous ketamine in emergencies requiring procedural sedation in children, as well as training and evaluating staff competencies in performing this procedure, are the aims of this study. Senior and mid-level physicians, including pediatric nurses working in the children's emergency department (ED), have easy access to this concise repository for carrying out this procedure safely and effectively.

The main objective was to implement a policy (guideline) for sedation in the children's ED. The secondary objectives were to determine whether the introduction of this policy increased the overall satisfaction rating of procedural sedation as a service offered in the Trust, to close skill gaps among staff through targeted training, and to assess the degree of recommendation for this ongoing standard of practice, in line with the current best evidence.

A retrospective review of pediatric sedation cases with fractures in the children's ED of Buckinghamshire Healthcare National Health Service (NHS) Trust was conducted between June 2022 and August 2023. Surveys were sent out to assess attitudes, sedation skills, and compliance with the Royal College of Emergency Medicine (RCEM) guidelines. A policy for pediatric sedation with intravenous ketamine was later created and put into effect. Pre- and post-intervention data were compared to assess changes in sedation competency, compliance, and confidence.

Out of the 103 pediatric fracture cases reviewed, 16 were identified as needing reduction upon initial evaluation, based on the child's condition at presentation. However, only one (6%) was treated with ketamine sedation in the children's ED, whereas nine cases (56%) were reduced under general anesthesia. The remainder was treated with varying use of nitrous oxide gas (Entonox) and diamorphine. Significant progress had been made after a clinical guideline for intravenous ketamine-assisted sedation in children was put into practice. Physicians reported improved knowledge of sedation techniques, knowledge of airway control, and safety in administering ketamine sedation. The percentage of respondents supporting the guidelines increased from 61% before the intervention (nValue=11) to 94% after (nValue​=17), using various metrics.

These results demonstrated how well evidence-based policies and training can improve performance, competence, and safety. The use of procedural sedation in the children's ED increased and the need for general anesthesia was significantly reduced after a uniform pediatric ketamine sedation policy was implemented. Targeted training to address skill gaps increased employee confidence and adherence to best practices.

## Introduction

An essential part of emergency care for children is sedation, which makes it possible to safely and successfully perform unpleasant procedures such as fracture reduction, burn dressings, etc. Due to its safety profile and dissociative properties, Ketamine is generally recommended by the Royal College of Emergency Medicine (RCEM) for use in pediatric sedation. However, many emergency rooms continue to underutilize it, which often leads to an increased need for general anesthesia. This research examines the current pediatric sedation practice in the children’s emergency department (ED) of Buckinghamshire Healthcare Trust and applies a standardized guideline to maximize the use of intravenous ketamine for these procedures.

Accidental injury is a leading cause of morbidity and mortality in children [1]. Although the National Health Service (NHS) does not readily provide specific data on trends in pediatric ED visits in the UK, evidence from other developed countries shows that the number of children with injuries attending visits is increasing annually [2]. Children frequently have difficulty managing painful and disruptive injuries that call for several different therapeutic approaches. This can lead to postponed or canceled procedures, an increased risk of complications, prolonged ED stays, and negative experiences for the child and their families. It might also lead to increased medical expenses because of the incurred costs from utilizing operating room procedures and general anesthesia. When clinically appropriate, the use of safe and effective sedation should be prioritized in order to minimize suffering, shorten hospital stays, and improve patient and caregiver satisfaction.

## Technical report

Methodology

This quality improvement project (QIP) was conducted in two phases.

Audit Phase

This is a retrospective review of pediatric procedural sedation cases from June 2022 to June 2023. Survey 1 was the baseline pre-intervention survey conducted using online questionnaires and discharge summaries of pediatric patients with clinical fractures between June 2022 and June 2023.

Intervention Phase

Development and implementation of a pediatric ketamine sedation policy, staff training, and a post-intervention survey between June 2023 and November 2024. In June 2023, Survey 2 surveyed 18 healthcare professionals working in the pediatric ED to assess knowledge, training, and competency for pediatric sedation. This heralded the beginning of the QIP's Plan-Do-Study-Act (PDSA) cycle. Survey 3 was conducted in November 2024, one month after the ketamine sedation guideline was released for use. After one month of implementation, training, and use of the guideline, Survey 3 assessed professional satisfaction, buy-in for subsequent cohorts, and skills gaps, and determined the extent to which the guideline improved overall understanding and confidence regarding pediatric procedural sedation.

Data Collection

Patient records specific to fracture presentations were reviewed to identify sedation cases, methods used, and outcomes. Questionnaires assessing clinical staff's confidence, competence, and adherence to the RCEM's best practice guidance were generated and sent as surveys, before and after the implementation of the new guideline. All surveys were created on Microsoft Survey Forms and circulated virtually to the clinicians working at the time of the survey through departmental media work groups (WhatsApp) and emails. This allowed for the data to be easily collected and meant it was easier for respondents to respond at a time that was convenient to them. The data generated was collated and computed with Excel Spreadsheets software (Microsoft Corp., Redmond, WA, USA) to generate results.

From the results of Survey 2, it became clear that a clinical guideline with established standards of practice (SoP) was urgently needed to ensure the smooth implementation of procedural sedation. Following the completion of the Survey 1 audit, the QIP department at Buckinghamshire Healthcare NHS Trust registered this project and granted full approval. The contents of the Ketamine Sedation Brochure (the practical derivative of the policy for use as an on-site procedural protocol) were compiled and organized by the authors, before review by the Trust's stakeholders (a multi-disciplinary network of senior doctors and clinical staff from the Critical Care, Pediatrics, Emergency Medicine and Anesthesia departments).

A medicine review form (this is the Trust's requirement for all clinical policies involving the use of medicines in children) was completed by the pharmacy department and the QIP project manager carried out an equality impact assessment survey (another requirement for large-scale improvement projects involving the care of minorities and vulnerable groups). After several editorial revisions, the completed project was submitted to the clinical guidelines team for upload to the authorized hospital's intranet hub. A succession of workshops, practical exercises, and ongoing improvements were delivered alongside paper versions of the ketamine sedation brochure to ensure harmony in implementing the new protocol.

Audience

We hoped for a perfect response rate but received varied outcomes from the sample cohort of senior (consultants), mid-level clinicians (intermediate and higher specialty residents and advanced care practitioners), as well as the pediatric nurses working in the children's ED. Each survey was anonymous. Respondents in Surveys 2 and 3 were the same individuals and belonged to the same departmental cohort. However, respondents for Survey 1 were from an earlier cohort, as departmental changes were modified by ongoing junior doctor training programs which ensured rotation of resident doctors to other hospitals in the UK.

Ethical Considerations

The draft of the guideline and ketamine sedation brochure (booklet) were formally submitted to the separate clinical governance teams of the ED and pediatric department following the completion of survey 2. The QIP was well received, and approvals were obtained to facilitate official inclusion in the Trust's intranet hub for hospital guidelines. There were no conflicts of interest. All documents were thoroughly reviewed and supported by appropriate references before being submitted to the clinical guidelines team.

Results

**Figure 1:**
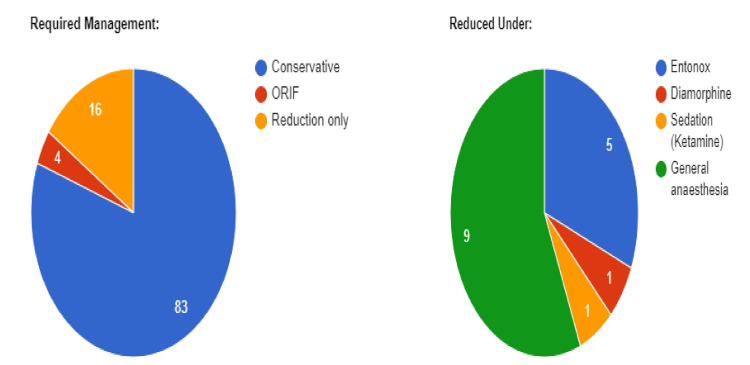
One hundred three fracture cases reviewed in the retrospective audit The first pie chart (labeled “Required Management”) showed that on the first fracture presentation: 16 cases were deemed to require reduction, 83 cases were managed conservatively (Splint or Bandage), and four cases required Open Reduction and Internal Fixation (ORIF). The second pie chart (labeled “Reduced Under”) showed that out of the 16 cases requiring reduction: one (6%) was performed under Ketamine, one (6%) was performed under Diamorphine only, five (31%) were performed under Entonox only, and nine (56%) were performed under general anesthesia in theater.

In total, we examined 103 pediatric fractures. Ketamine sedation was used in the ED in only one case, while 56% of cases required treatment in the operating room under general anesthesia (Figure 1).

Pre-intervention

**Figure 2:**
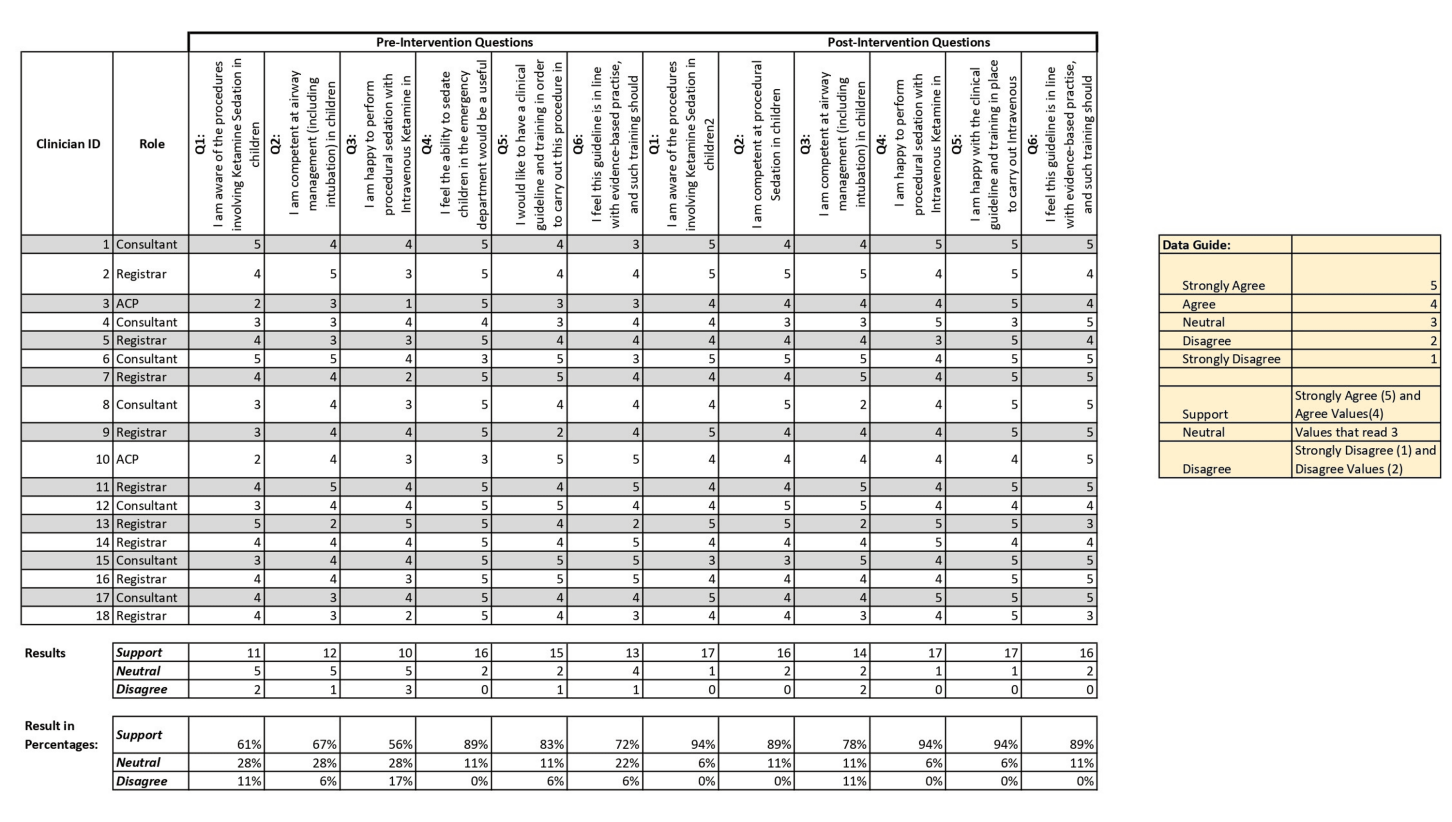
Survey 2 and 3 questions compared before and after introduction of guidelines Surveys 2 and 3 both comprised six questions for correlation.

According to data from surveys conducted before the introduction of the clinical guideline (pre-intervention phase), physicians' confidence and skill in using ketamine for pediatric sedation varied. Only 61% of respondents reported awareness of the steps involved in ketamine sedation in children, 56% of respondents felt competent in airway management, including intubation, 61% of respondents said they were confident in using ketamine for procedural sedation, and 83% of respondents reported a need for a structured clinical guide to standardize practices (Figure 2).

Post-intervention Survey Results

Once the clinical guideline and associated training were put into practice (post intervention), there was significant progress across all metrics. Firstly, procedural awareness has increased significantly. Additionally, 94% of respondents expressed confidence in their ability to competently manage the airway, an increase of 38% compared to pre-procedure levels. In addition, confidence in administering sedation increased significantly as 89% of respondents believed the guideline reflected evidence-based practice and supported its continued use (Table 1).

**Table 1:** Changes in responses before and after the guideline (intervention) was introduced. Q: Question

Result summary table		
Survey questions	Pre-intervention survey responses (%)	Post-intervention survey responses (%)
Q1	61	89
Q2	67	78
Q3	56	94
Q4	89	94
Q5	83	89
Q6	72	89

**Figure 3:**
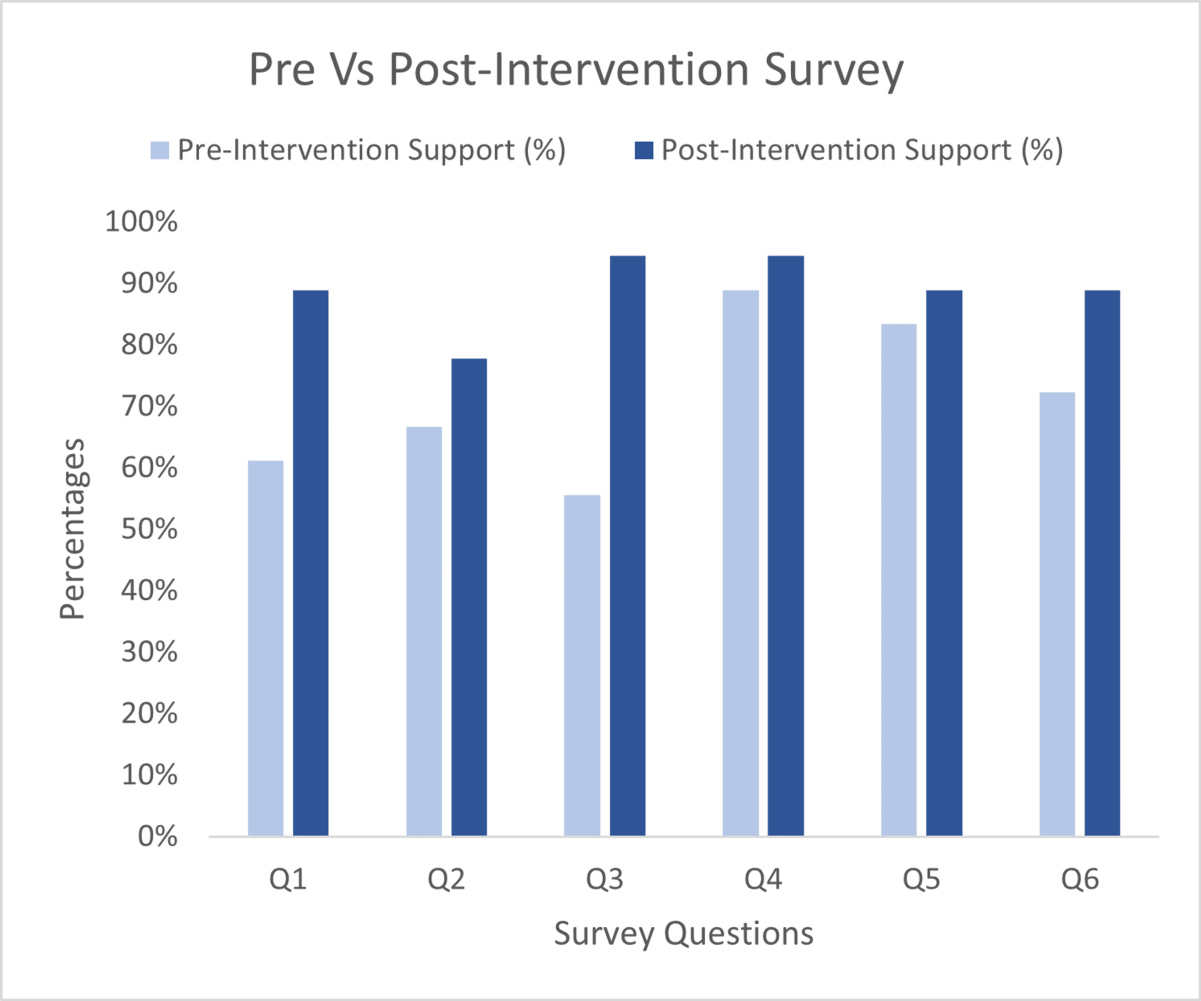
Graphs comparing survey responses before and after establishing intravenous Ketamine guideline

Graphical representations showed the significant impact of change with the introduction of the guideline (Figure 3).

## Discussion

Conscious sedation is a monitored altered state of consciousness that can be used instead of general anesthesia to perform brief therapeutic, dental, or diagnostic procedures on nervous individuals or patients with compromised health [2]. It enables the therapeutic procedure to be carried out with as little physical and mental stress as possible. In order to maintain communication with the patient, who simultaneously responds to commands and can keep the airway open, drugs are used to induce a state of central nervous system (CNS) depression (but not unconsciousness) that is strong enough to withstand the procedural trespass. Conscious sedation differs from anesthesia in one respect: it is safer because the protective airway reflexes are preserved [3].

The pediatric age group in the UK is generally defined as those between the ages of one and 18, with divisions according to developmental stages and age to efficiently customize care and services. Sedation in the pediatric ED is primarily used to protect the child's health while enabling the safe execution of procedures. By promoting drowsiness or sleepiness, sedation reduces physical discomfort, anxiety, fear, and physiological trauma. Along with reducing hospital stays, it can enhance the experience for the child's parents or other caregivers. Sedation also enables prompt and safe discharge, eliminating the need for extended medical monitoring, and it lowers operating room expenses by avoiding general anesthesia.

Ketamine is ideal for procedural sedation due to its effectiveness and proven safety profile [2,3]. Instead of the reticular activating system, which crucially controls consciousness and wakefulness, the main site of action of ketamine is in the cortex and subcortical regions of the CNS [3-7]. It causes what is known as “dissociative sedation,” which is typified by substantial pain relief, immobility, amnesia, light sleep, and a feeling of detachment from the body and environment. Muscle tone increases, respiratory reflexes are preserved, breathing is not slowed, limb movements occur, and eyes may remain open and glassy. Intravenous administration of ketamine is preferred because of its rapid onset of action, titratability, shorter recovery time, improved control over sedation depth and side effects, and its perception of being less traumatic, as an intravenous line is more likely to be placed already in controlled hospital settings, thus avoiding the need for additional injections.

Effective intravenous sedation can last up to twenty minutes, with a typical clinical onset time of one minute. Shortly thereafter, the patient begins to recover but may remain amnestic for one to two hours. The drug has an elimination half-life (t½) of three to four hours, after which it is metabolized in the liver. Interestingly, children are more tolerant of ketamine than adults [7].

Side effects of ketamine sedation according to data from RCEM include nausea and vomiting (5%-10% of children may vomit during the recovery period from sedation); aspiration; hypotension; mild restlessness (which usually does not require treatment), and emergence phenomena. Other rare, documented side effects include hypoxia (simple repositioning of the airway is usually sufficient to resolve this problem); apnea (prevalence of 0.3%), and laryngospasm (a rare, transient event with 0.02% reported incidence of intubation-related laryngospasm) [3-5]. Contraindications for ketamine include abnormal airways (which may impede ventilation); active upper respiratory tract infection; previous adverse reaction to ketamine; previous complications with ketamine sedation; depressed level of consciousness; situations where definitive treatment imminently requires general anesthesia, and in procedures involving a body part that requires direct access to the airway [3-6].

Staff and training 

Prescribing and dispensing sedation must be performed by appropriately trained senior physicians and mid-level residents with pharmacological knowledge and understanding of procedural sedation [4,5]. ED consultants will provide regular training to ensure appropriate credentialing before individuals are expected to deliver this procedure unsupervised. Through training, staff will be able to demonstrate expertise in the following areas: administering selected sedation techniques; assessing the child's monitoring needs and use of necessary equipment; observation of clinical signs and management of complications; monitoring recovery and implementing advanced pediatric life support skills to manage adverse events. To maintain their current skills, sedation professionals will undergo regular continuous professional development, as well as timely renewal of their advanced pediatric life support (APLS) certifications.

Ketamine sedation booklet

This brochure provides the detailed procedure for the seamless use of intravenous ketamine for pediatric sedation in the children's ED. According to the Royal College of Anesthetists and RCEM, the ED clinician performing sedation is referred to as “the sedationist” and is required to complete the ketamine sedation booklet at each phase of the ED process that involves the procedure [5]. The sedationist must conduct a pre-assessment of the child and document all findings in accordance with the booklet before deciding to administer procedural sedation, taking into account pertinent departmental and nursing factors.

When determining whether sedation is appropriate, it is important to consider the child's developmental and psychological state, health, surgical history, medications, allergies, past ketamine sedation exposure, side effects, and recovery. The on-call anesthetist should be consulted if the pre-assessment indicates the need for specialist intervention. The sedationist must verbally inform the parent/caregiver of the risks and benefits of the procedure, available alternative treatments, and side effects of sedation. The oral or written consent of the parent or carer must be noted in the sedation booklet.

The sedationist must also complete the pre-procedure checklist as stated. Before ketamine administration, patients must undergo baseline monitoring carried out by the nurses assigned to the procedure. The sedation booklet contains the following vital signs: blood pressure, heart rate, respiratory rate, oxygen saturation, and end-tidal carbon dioxide. Side effects encountered during the procedure, as well as treatments given, must be documented in the appropriate columns provided in the booklet.

Preparing the child

Psychological preparation is necessary to manage behavioral problems and reduce the child's anxiety. When preparing older children, it can be helpful to explain the process, the feelings they may be experiencing, and the roles of all staff. Psychological interventions for younger children such as play therapy and distraction techniques may be used when appropriate [8]. To reduce separation anxiety, the presence of parents and caregivers is recommended during the procedure [9]. Fasting is not required for this procedure. According to the joint policy statement of the American College of Emergency Physicians (ACEP) and the Emergency Nurses Association (ENA), nothing by mouth (NPO) status has been shown to have no influence on the risk of aspiration or vomiting during emergency sedation [4]. Current evidence also shows that the rate of gastric aspiration remains independent of whether a child is fasting or not [10-14]. In addition, aspiration of stomach contents into the lungs rarely occurs in children. Therefore, the clinical consequences of clear fluid aspiration are rarely severe enough to require long-term monitoring or occasionally antibiotic therapy, regardless of the liquid fast regimen [10-16].

The ED discharge criteria section of the sedation booklet must be completed prior to possible discharge following the procedure. The child's observation skills should be stable after the procedure and remain within the intended age and weight ranges. Advice on safety nets is to be provided, and children must be placed with responsible adults at home. The dosages of all medications listed in the guidelines are taken in accordance with RCEM recommendations and the British National Formulary for Children (Table 2) [17].

**Table 2:** Summary of guideline medications depicting mechanism of action and dosages NMDA: N-methyl-D-aspartate, GABA: Gamma-aminobutyric acid, 5HT: 5-Hydroxytryptamine receptor 3, μ: Mu (Greek letter), κ: Kappa (Greek letter), δ: Delta (Greek letter)

Drugs	Mechanism of action	Dose/route
Ketamine	NMDA and Glutamate receptor antagonist. Partial opiate µ-receptor agonist in the cortex and sub-cortical areas of the brain	Intravenously: 0.5-1mg/kg bolus, with 0.5mg/Kg top-ups (if required), every 5-10 minutes
Intranasal Diamorphine	Opiate μ, κ, and δ receptor agonist	Intranasally (aged 2-15) given by body weight. 12-17kg: 1.44mg for one dose; 18-23kg: 2.16mg for one dose; 24-29kg: 2.88mg for one dose; 30-39kg: 3.2mg for one dose; 40-50kg: 4.8mg for one dose.
Ondansetron	5HT3 receptor antagonist	Intravenously: 100 micrograms/kg for 1 dose (maximum per dose is 4mg), to be given over at least, 30 seconds.
Midazolam (rarely used)	GABA agonist	Discuss with senior clinician before use.

## Conclusions

This QIP highlights the underuse of ketamine for pediatric sedation in emergencies and identifies staff confidence as a key barrier. Through the successful implementation of a standardized guideline and the provision of targeted training, ketamine use and physician confidence increased, demonstrating that primary and secondary objectives were met. These findings illustrate the significance of incorporating evidence-based guidelines into clinical practice to enhance procedural confidence, competence, and ultimately patient safety. Furthermore, this study supports the scalability of such interventions to other areas that need similar standardization. Future projects should focus on sustaining these improvements and exploring broader applications of the guideline in similar healthcare settings, especially regarding patient outcomes and resource utilization in the ED.

## Figures and Tables

**Figure 4:**
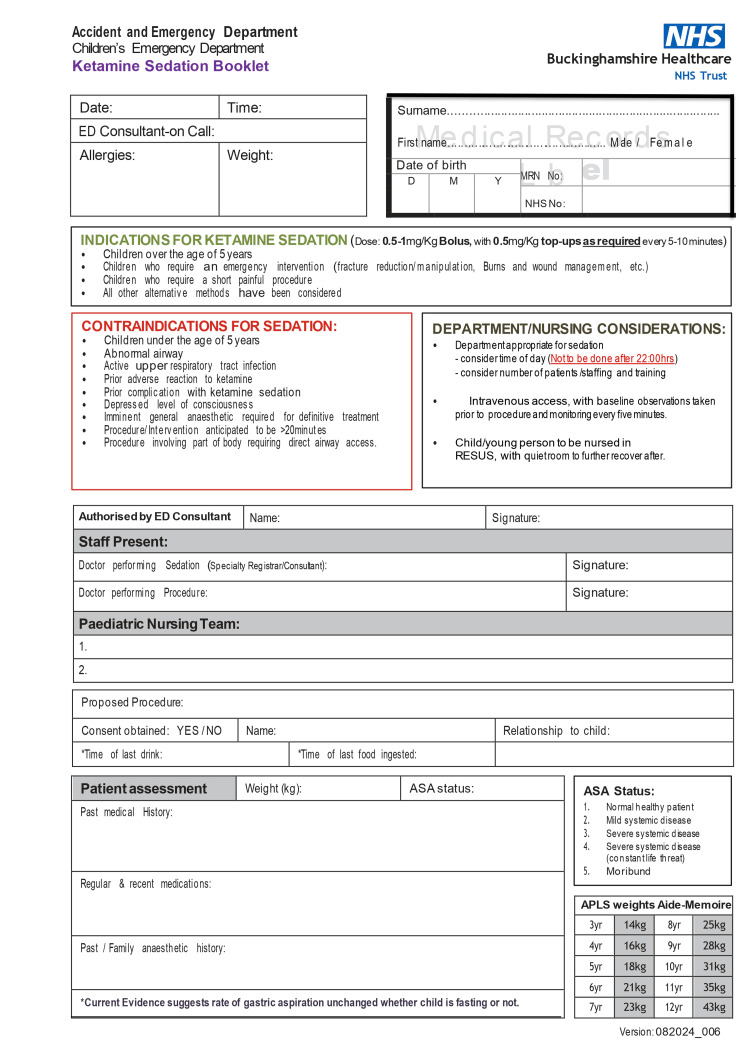
Ketamine sedation booklet (Page 1)

**Figure 5:**
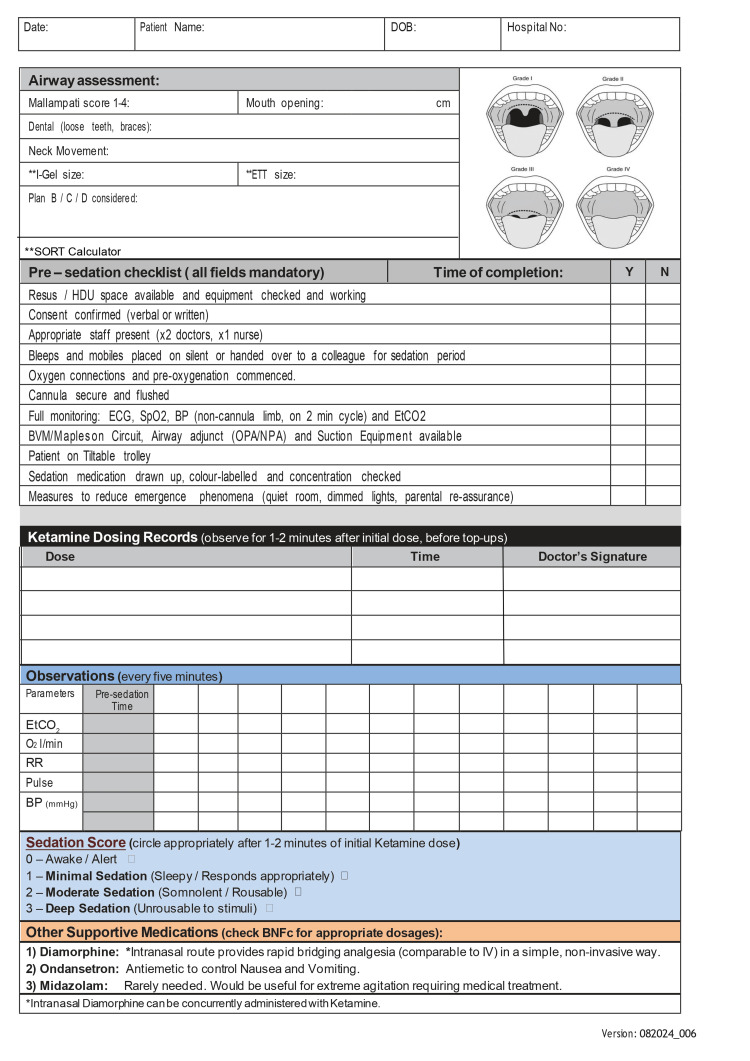
Ketamine sedation booklet (Page 2)

**Figure 6:**
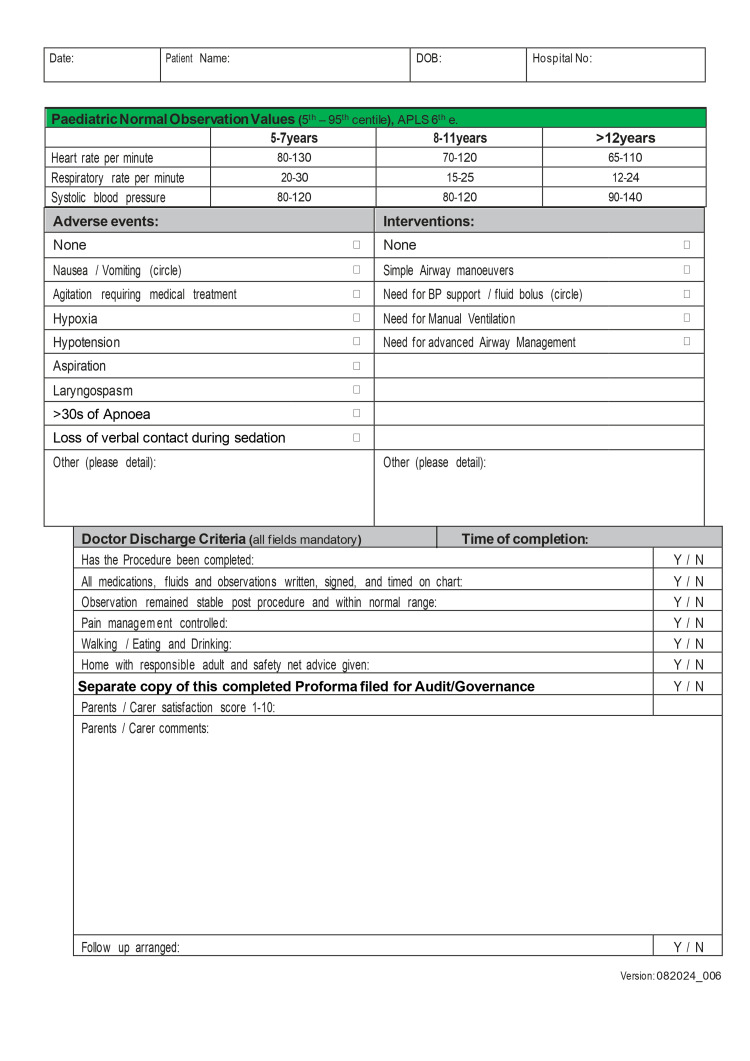
Ketamine sedation booklet (Page 3)
